# Pan2‐Pan3 Complex‐Mediated Deadenylation Enforces mRNA Quality Control for Infection of the Rice Blast Fungus

**DOI:** 10.1002/advs.202518269

**Published:** 2026-01-20

**Authors:** Ziwei Lv, Junting Feng, Shenxian Zhou, Zhiguang Qu, Jun Peng, Xiao‐Lin Chen, Deng Chen

**Affiliations:** ^1^ Environment and Plant Protection Institute Chinese Academy of Tropical Agricultural Sciences Haikou China; ^2^ State Key Laboratory of Agricultural Microbiology Provincial Key Laboratory of Plant Pathology of Hubei Province College of Plant Science and Technology Huazhong Agricultural University Wuhan China; ^3^ Institute of Tropical Bioscience and Biotechnology Chinese Academy of Tropical Agricultural Sciences Haikou China; ^4^ Department of Agricultural Biotechnology Seoul National University Seoul South Korea

**Keywords:** deadenylation, fungal infection, mRNA decay, Magnaporthe oryzae, Pan2‐Pan3 complex

## Abstract

Poly(A) tail shortening by deadenylases is a central checkpoint linking mRNA fate to eukaryotic development, yet its impact on fungal pathogenesis remains unexplored. Here, we uncover that the Pan2‐Pan3 deadenylase complex is a master regulator of infection in the rice blast fungus *Magnaporthe oryzae*. Pan2 and Pan3 form a catalytically active complex that localizes to P‐bodies and globally trims poly(A) tails to enforce mRNA quality control. Deletion of either or both subunits abolishes this quality‐control checkpoint, causing severe virulence loss due to arrested appressorium maturation, disrupted glycogen/lipid mobilization, and impaired autophagy. Integrating poly(A)‐seq and transcriptome profiling reveals 390 mRNAs whose poly(A) tails are ≥ 5 nt longer and whose steady‐state levels are elevated in the Δ*pan2*Δ*pan3* mutant; among them, *ATG5*, *GLS2*, and *DES1*—key genes governing autophagy, ER quality control, and ROS detoxification respectively—are directly deadenylated by Pan2‐Pan3. Loss of deadenylation destabilizes these mRNAs and reduces their protein output, thereby crippling infection. Our findings establish Pan2‐Pan3 complex‐mediated deadenylation as an essential post‐transcriptional layer that orchestrates fungal virulence through stringent mRNA quality control, offering a novel target for crop protection.

## Graphical abstract

Discovery that deadenylation, rather than transcription, acts as the rate‐limiting step for developmental timing in a plant pathogen. Evidence that P‐body integrity and mRNA decay are mechanistically coupled to rapid cellular differentiation under environmental stress. Identification of Pan2‐Pan3 as a pathogen‐specific “meta‐virulence factor” absent in plants, offering a highly specific target for sustainable crop protection.

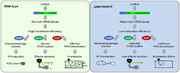

## 1 Introduction

The journey of an mRNA from birth to decay is punctuated by reversible modifications that dictate when, where, and how efficiently it is translated. Among these, the non‐templated poly(A) tail appended to the 3′ end of eukaryotic transcripts is both a shield and an hourglass: while its length initially protects mRNA from exonucleases and promotes translation, its gradual shortening by deadenylases ultimately licenses irreversible silencing [1, 2]. Two conserved engines—the Ccr4‐Not and Pan2‐Pan3 complexes—execute this temporal program, yet their potential to act as environmentally responsive checkpoints has been dissected almost exclusively in yeast and mammalian cells [3, 4, 5]. In the first phase, the Pan2‐Pan3 complex, which comprises the catalytic subunit Pan2, a member of the RNase D family, and the regulatory subunit Pan3, promotes the removal of distal adenosines (A) to proper length [6, 7]. In the second phase, the Ccr4‐Not complex, which consists of two catalytic subunits including the Ccr4 (Carbon catabolite repressor 4) and Pop2 (PGK promoter directed overproduction), is considered to shorten the poly(A) tail to oligo (A) at the proximal in the second phase of deadenylation [8, 9]. Pan2‐Pan3 initiates deadenylation, trimming the nascent poly(A) tract to an intermediate length, thereby setting the tempo for subsequent decay or storage [10, 11]. Although this biochemical sequence has been dissected in yeast and mammalian cells, its biological significance in filamentous fungi—and particularly in plant pathogens—remains almost entirely unexplored. Whether a single deadenylase complex can serve as a universal timer that quantifies external cues and allocates developmental time remains unknown.

*Magnaporthe oryzae*, the rice blast fungus, is an ideal model with which to test this hypothesis [12]. *M. oryzae* is among the most destructive pathogens on earth, responsible for losses that could feed hundreds of millions of people annually [13, 14]. Its success depends on a single, time‐critical decision: within hours of landing on a leaf, the fungus must convert surface hydrophobicity and nutrient scarcity into a melanized appressorium that generates enormous turgor to breach the host cuticle. Formation of this structure is accompanied by wholesale metabolic remodeling: glycogen and lipid reserves are mobilized, autophagy is triggered, reactive oxygen species are detoxified, and secreted effectors are deployed to suppress host immunity [15, 16, 17, 18]. Each of these processes is driven by waves of gene expression whose timing must be exquisitely coordinated; yet the post‐transcriptional mechanisms that enforce such coordination are unknown. Intriguingly, the *M. oryzae* genome encodes clear orthologues of Pan2 and Pan3, raising the possibility that deadenylation functions not merely as a housekeeping process, but as an RNA‐based countdown timer that synchronizes metabolic remodeling, autophagy, and host penetration.

Here, we investigate whether Pan2‐Pan3 complex‐mediated poly(A) tail removal is a decisive checkpoint that synchronizes the molecular events required for appressorium maturation and host penetration. By combining genetics, high‐resolution imaging, global poly(A)‐tail profiling, and plant infection assays, we reveal that the complex is indispensable for virulence. Loss of *PAN2* or *PAN3* disrupts the temporal decay of key transcripts governing autophagy, endoplasmic‐reticulum quality control, and oxidative stress tolerance, thereby arresting infection at the very moment the fungus attempts to enter the plant. These findings position Pan2‐Pan3 as a master regulator that translates environmental cues into precisely timed gene expression, and they establish deadenylation as a novel, pathogen‐specific target for sustainable crop protection.

## 2 Results

### 2.1 Identification of the Pan Complex in *Magnaporthe oryzae*

**FIGURE 1:**
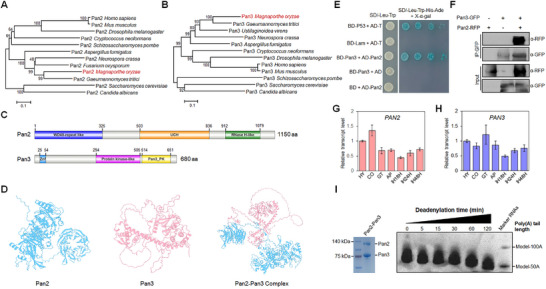
Characteristics and interaction of Pan2 and Pan3. A) Phylogenetic analysis of Pan2 across model eukaryotes. B) Phylogenetic analysis of Pan3 across model eukaryotes. C) Domain architecture of Pan2 and Pan3 proteins. D) Predicted protein structures of Pan2, Pan3, and the Pan2‐Pan3 complex. For the structure‐based Pan2 and Pan3 interaction prediction, ipTM = 0.38, pTM = 0.46. E) Yeast two‐hybrid (Y2H) assay confirming the interaction between Pan2 and Pan3. The experiment was conducted with three independent replicates. F) Co‐immunoprecipitation (co‐IP) assay validating the Pan2‐Pan3 interaction. The experiment was conducted with three independent replicates. G,H) Expression profiles of *PAN2* (G) and *PAN3* (H) at various developmental stages: hyphae (HY), conidia (CO), germ tubes (GT), appressoria at 12 hpi (AP), invasive hyphae at 18 hpi (IH18H), 24 hpi (IH24H), and 48 hpi (IH48H). Expression in HY was set to 1. Error bars represent SD of three biological replicates. I) In vitro deadenylation of a synthetic yeast 90A RNP substrate by the Pan2‐Pan3 complex, showing a phased poly(A) tail distribution. The left panel shows the loaded, purified Pan2‐Pan3 fusion protein visualized by Coomassie blue staining. The assay was performed with three independent biological replicates.

Through a search for Pan orthologous proteins in the NCBI database, we identified two subunits—Pan2 (MGG_17449) and Pan3 (MGG_02939)—in *M. oryzae*. Phylogenetic analysis revealed that both proteins are highly conserved among model eukaryotes, particularly plant pathogenic fungi. Pan2 shares high similarities with its homologs in *Gaeumannomyces tritici* and *Fusarium oxysporum* (Figure 1A), while Pan3 is closely related to homologs in *G. tritici* and *Ustilaginoidea virens* (Figure 1B).

*PAN2* encodes a 1150‐amino acid protein containing an N‐terminal WD40‐repeat‐like domain, a ubiquitin C‐terminal hydrolase (UCH) domain, and an RNase H‐like motif near the C‐terminus. The Pan3 protein is predicted to comprise 680 amino acids with a CCCH‐type zinc finger domain, a protein kinase‐like motif, and a C‐terminal pseudokinase domain. Notably, the two proteins share no sequence similarity (Figure 1C). To determine whether they form a deadenylation complex, we used AlphaFold to predict their 3D structural alignment. This analysis suggested a high possibility of complex formation, indicating a potential interaction between Pan2 and Pan3 (Figure 1D).

To test this hypothesis, we conducted yeast two‐hybrid assays. Yeast strains co‐transformed with BD‐Pan3 and AD‐Pan2 vector grew on SD/‐Leu‐Trp‐His‐Ade medium and produced blue coloration with X‐α‐Gal (Figure 1E). Additionally, co‐immunoprecipitation (Co‐IP) assays using RFP‐tagged Pan2 and GFP‐tagged Pan3 detected an RFP signal after precipitation with GFP beads (Figure 1F). These results confirm that Pan2 interacts with Pan3.

To investigate subcellular co‐localization, we constructed vectors expressing Pan2‐RFP and Pan3‐GFP in the wild‐type (WT) strain P131. As expected, merged fluorescence signals were observed in hyphae (HY), conidia (CO), appressoria (AP), and invasive hyphae (IH), indicating Pan2‐Pan3 co‐localization (Figure S1A,B). Collectively, these results establish that Pan2 and Pan3 interact to form a functional complex.

To explore their biological functions, we analyzed *PAN* gene expression across developmental stages (HY, CO, GT, AP, IH18H, IH24H, IH48H). Both *PAN2* and *PAN3* showed relatively high expression in vegetative hyphae, conidia, and germ tubes, suggesting roles in asexual development. Moreover, their expression increased progressively during infection (from IH18H to IH48H), indicating involvement in pathogenesis (Figure 1G,H).

Given their predicted specificity as poly(A)‐targeting ribonuclease, we assessed the deadenylation activity of the Pan2‐Pan3 complex in vitro. In a deadenylation assay, exogenous addition of purified recombinant Pan2‐Pan3 protein degraded 90A mRNA (Figure 1I), demonstrating that the complex exhibits deadenylation activity and likely mediates poly(A) removal during mRNA decay.

### 2.2 The Pan2‐Pan3 Complex is Essential for Asexual Development and Morphogenesis in *M. oryzae*

To further investigate the molecular function of *PANs*, we generated knockout mutants of *PAN2* and *PAN3* separately in strain P131 using a split‐PCR strategy (Figure S2A). Two *PAN2* deletion mutants and two *PAN3* deletion mutants were identified by PCR (Figure S2B,C). The mutants *pan2*‐1 (genetic name Δ*pan2*) and *pan3*‐1 (genetic name Δ*pan3*) were used in subsequent experiments. Since Pan2 and Pan3 form the deadenylation complex, we simultaneously deleted both genes to assess the complex's role in *M. oryzae*. Using a similar method, *PAN3* was deleted in the Δ*pan2* background (Figure S2D). PCR identification yielded two double‐deletion mutants (Figure S2E), one of which, *pan2pan3‐*1 (genetic name Δ*pan2*Δ*pan3*), was selected for further study. Additionally, we generated complementary strains for Δ*pan2* and Δ*pan3* by transforming each single‐deletion mutant with its respective native promoter‐driven coding region (Figure S3A). Three randomly picked Δ*pan2* complementary transformants (Δ*pan2*/*PAN2*‐1, ‐2, and ‐3) and three Δ*pan3* complementary transformants (Δ*pan3*/*PAN3*‐1, ‐2, and ‐3) were verified by PCR (Figure S3B). The complementary strains exhibited vegetative growth and pathogenicity phenotypes that recovered to WT levels (Figure S3C,D). Strains Δ*pan2*/*PAN2*‐1 (hereafter referred to as Δ*pan2*/*PAN2*) and Δ*pan3*/*PAN3*‐1 (hereafter named Δ*pan3*/*PAN3*) were used for subsequent assays.

To determine if PANs affect asexual development in *M. oryzae*, we inoculated WT, Δ*pan2*, Δ*pan2/PAN2*, Δ*pan3*, Δ*pan3/PAN3*, and Δ*pan2*Δ*pan3* strains on oatmeal tomato agar (OTA). After 5 days of culture, Δ*pan2* and Δ*pan3* colonies exhibited significantly smaller diameters than WT, Δ*pan2/PAN2*, or Δ*pan3/PAN3*. Vegetative growth of Δ*pan2*Δ*pan3* was slower than that of every single‐deletion mutant (Figure S4A,B). We then stained hyphal tips with calcofluor white (CFW). As expected, hyphal cell length in the *PAN2* or *PAN3* single‐deletion mutants was significantly shorter than in WT or the complementary strains, but longer than in the double‐deletion mutant (Figure S4C,D). Interestingly, vegetative growth and colony morphology were similar between Δ*pan2* and Δ*pan3*. Observation of conidiophores revealed numerous conidia on each conidiophore of Δ*pan2*, Δ*pan3*, and Δ*pan2*Δ*pan3*. In contrast, WT, Δ*pan2*/*PAN2*, and Δ*pan3*/*PAN3* produced far fewer spores per conidiophore. This observation was consistent with conidiation quantification results (Figure S4E), which showed that Δ*pan2*, Δ*pan3*, and Δ*pan2*Δ*pan3* produced significantly more conidia than the WT or complementary strains (Figure S4F). Together, these results indicate that both *PAN2* and *PAN3* are important for asexual development in *M. oryzae*.

Given the reduced vegetative growth of *PAN* deletion mutants, we investigated their sensitivity to distinct stresses. We added cell wall integrity perturbing agents—Calcofluor white (CFW), Congo red (CR), and sodium dodecyl sulphate (SDS)—separately to complete medium (CM) plates. Both single‐ and double‐deletion mutants exhibited significantly higher growth inhibition ratios than WT. Furthermore, when the osmotic stress agent NaCl were added to CM, the mycelial growth inhibition rate of Δ*pan2*Δ*pan3* is higher than that of WT (*p* = 0.042), although no significant difference was detected between the single‐deletion mutants and WT. Conversely, under sorbitol treatment, the inhibition rate was higher for WT and the complementary strains compared to Δ*pan2* (Figure S5A,B). These results suggest *PAN2* and *PAN3* are important for cell wall integrity and may be involved in osmotic stress response.

### 2.3 Pan2‐Pan3 Complex Orchestrates Virulence in *M. oryzae*

**FIGURE 2:**
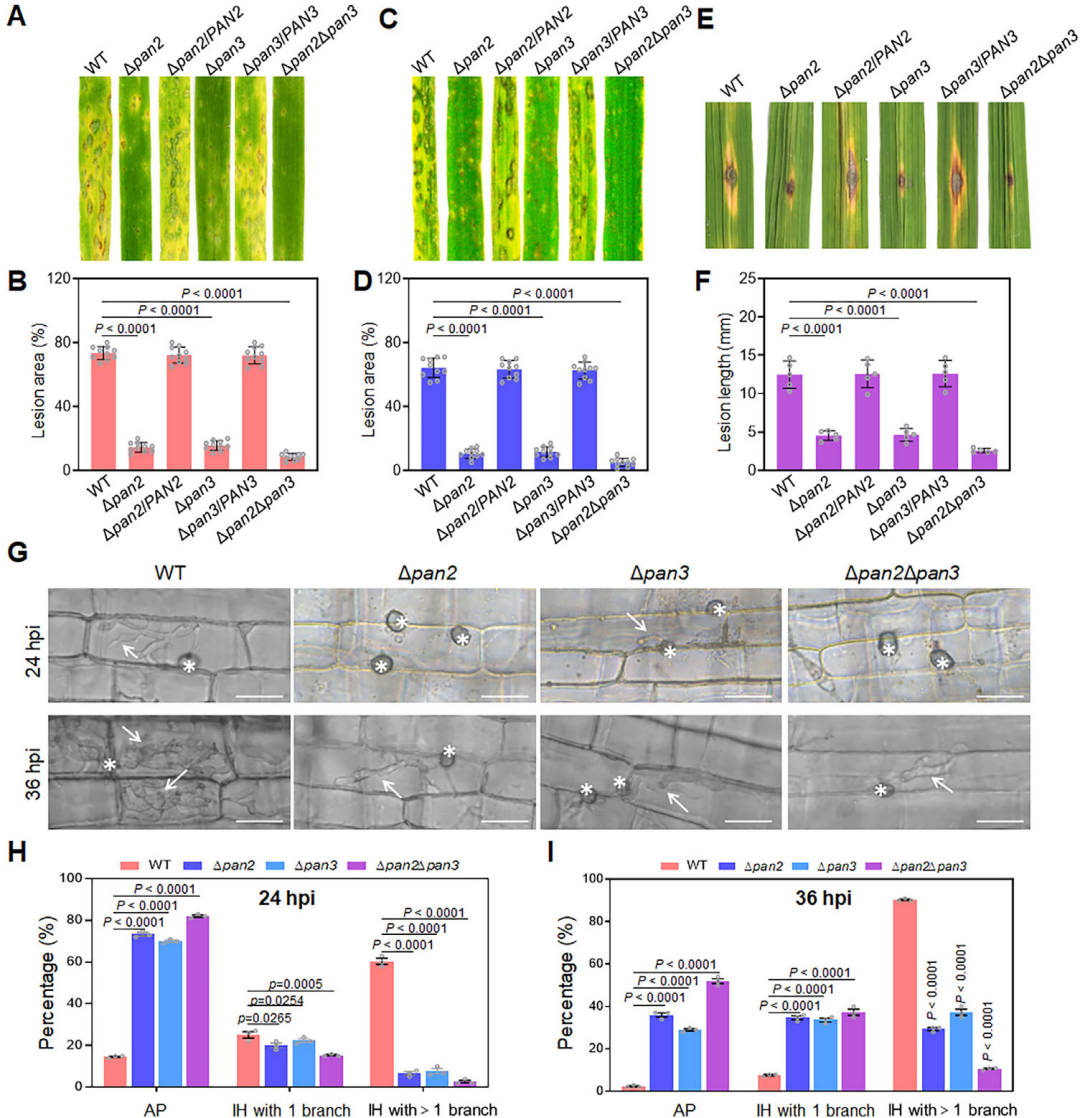
Pan2 and Pan3 are required for full virulence of *M. oryzae*. A) Symptoms on barley leaves spray‐inoculated with conidia of WT, Δ*pan2*, Δ*pan2*/*PAN2*, Δ*pan3*, Δ*pan3*/*PAN3*, and Δ*pan2*Δ*pan3*. B) Quantification of lesion area on inoculated barley leaves (one‐way ANOVA, Dunnett's test, *n* = 10, error bars = SD). C) Symptoms on rice seedlings spray‐inoculated with conidia of the indicated strains. D) Quantification of lesion area on rice seedlings (one‐way ANOVA, Dunnett's test, *n* = 10, error bars = SD). E) Symptoms on scratch‐wounded rice seedlings inoculated with conidia. F) Quantification of lesion length on rice seedlings (one‐way ANOVA, Dunnett's test, *n* = 10, error bars = SD). G) Invasive growth of WT, Δ*pan2*, Δ*pan3*, and Δ*pan2*Δ*pan3* in rice sheaths at 24 and 36 hpi. Asterisks indicate appressoria; arrows indicate invasive hyphae. Scale bar: 20 µm. H) Percentage of AP and IH at 24 hpi (one‐way ANOVA, Dunnett's test, three biological replicates, error bars = SD). For each replicate, a total of 50 appressoria or infection hyphae were randomly selected and counted. I) Percentage of AP and branched IH at 36 hpi (one‐way ANOVA, Dunnett's test, three biological replicates, error bars = SD). Fifty appressoria or infection hyphae were counted per replicate.

To investigate the roles of *PAN2* and *PAN3* in pathogenicity, we first assessed the virulence of *PAN2* and *PAN3* deletion mutants via spray inoculation on barley hosts. Compared to the WT and complementary strains, Δ*pan2* and Δ*pan3* mutants produced fewer and smaller lesions. Furthermore, the lesion area caused by the double mutant Δ*pan2*Δ*pan3* was even smaller than that caused by the single mutants (Figure 2A,B). These findings were further confirmed through spray inoculation on rice seedlings (Figure 2C,D).

To examine whether lesion extension was affected by Pan proteins, we performed scratch inoculations with the relevant strains. Lesions caused by WT, Δ*pan2/PAN2*, or Δ*pan3/PAN3* were significantly longer than those caused by Δ*pan2* or Δ*pan3*. Moreover, lesion expansion was severely restricted in rice leaves inoculated with Δ*pan2*Δ*pan3* (Figure 2E,F). The limited lesion extension on scratched leaves suggests impaired fungal invasive growth.

We therefore monitored the infection process of these strains in rice sheaths. At 24 h post‐inoculation (hpi), the proportion of appressoria (AP) that failed to develop into invasive hyphae (IH) was significantly higher in Δ*pan2*, Δ*pan3*, and Δ*pan2*Δ*pan3* compared to WT. Approximately 60% of AP in WT developed into IH with more than one branch, far exceeding that observed in the *PAN* deletion mutants (Figure 2G,H). By 36 hpi, the percentage of branched IH in the mutants remained markedly lower than in WT (Figure 2G,I). A similar infection process was observed in barley epidermis at 24, 30, and 42 hpi, and the results were consistent with those from rice sheaths (Figure S6A,B). These findings indicate that both *PAN2* and *PAN3* are required for invasive growth and play critical roles in the pathogenicity of *M. oryzae*.

### 2.4 Loss of Pan2‐Pan3 Impairs Appressorium Maturation

**FIGURE 3:**
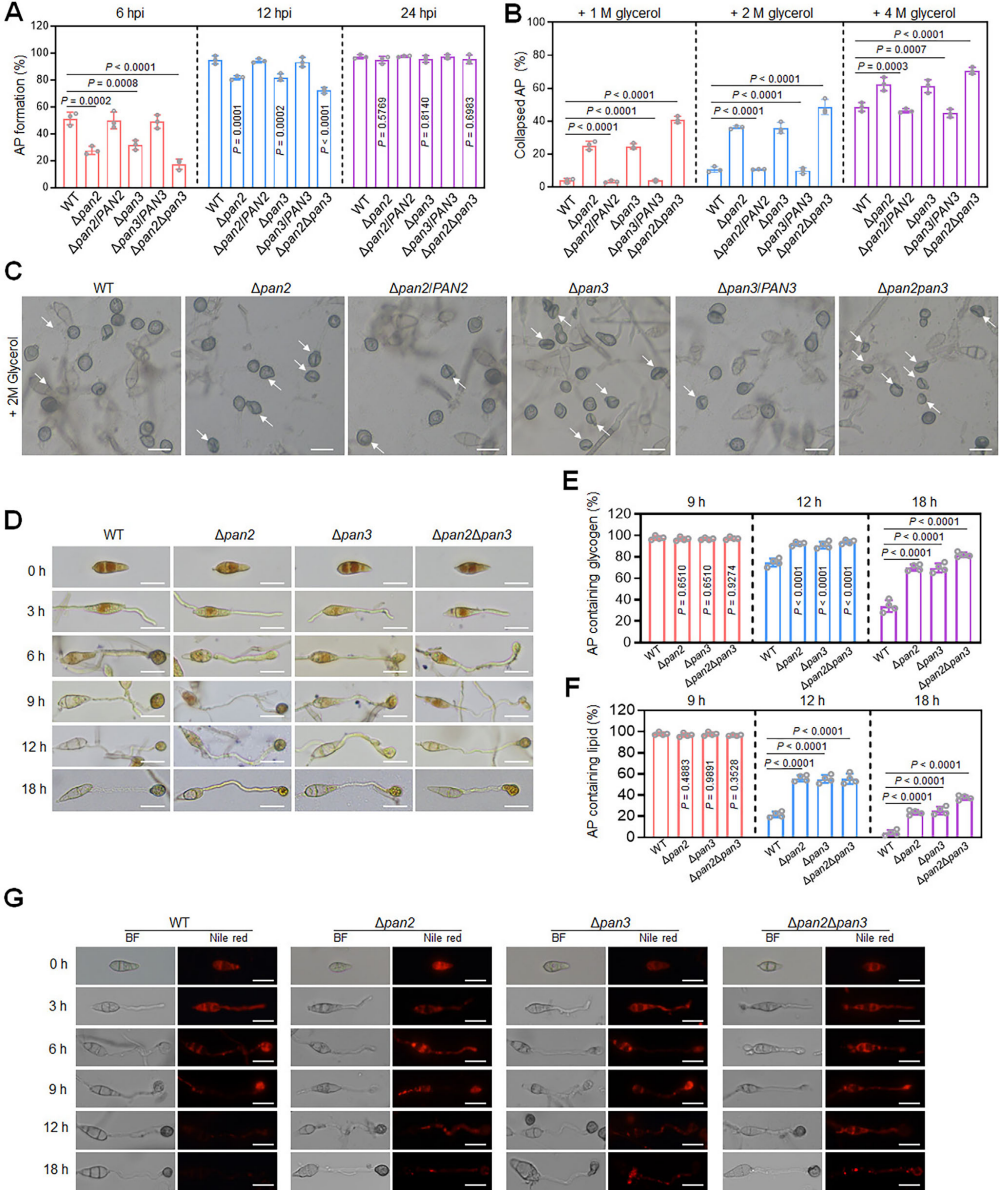
The Pan2‐Pan3 complex is essential for appressorium maturation. A) Appressorium formation rates in WT, Δ*pan2*, Δ*pan2*/*PAN2*, Δ*pan3*, Δ*pan3*/*PAN3*, and Δ*pan2*Δ*pan3* at 6, 12, and 24 hpi (one‐way ANOVA, Dunnett's test, three biological replicates, error bars = SD). For each replicate, a total of 50 conidia were randomly selected and counted. B) Percentage of collapsed appressoria after treatment with 1, 2, and 4 M glycerol (one‐way ANOVA, Dunnett's test, three biological replicates, error bars = SD). For each replicate, a total of 50 appressoria were randomly selected and counted. C) Collapsed appressoria following treatment with 2 M glycerol. Arrows indicate collapsed appressoria. Scale bar: 20 µm. D) Glycogen staining with KI/I_2_ during appressorium development in WT, Δ*pan2*, Δ*pan3*, and Δ*pan2*Δ*pan3* at the indicated time points. Scale bar: 20 µm. E) Percentage of appressoria containing glycogen (one‐way ANOVA, Dunnett's test, four biological replicates, error bars = SD). Fifty appressoria were counted per replicate. F) Percentage of appressoria containing lipids (one‐way ANOVA, Dunnett's test, four biological replicates, error bars = SD). 50 appressoria were counted per replicate. G) Lipid staining with Nile red during appressorium development. BF, bright field. Scale bar: 20 µm.

Since the appressorium is an essential infection structure of *M. oryzae*, we investigated whether appressorium formation or maturation was impaired in *PAN2*‐ or *PAN3*‐depleted strains. At 6 and 12 hpi, the appressorium formation rate of Δ*pan2* and Δ*pan3* was significantly lower than that of the WT and complementary strains, but higher than that of Δ*pan2*Δ*pan3*. By 24 hpi, however, no significant differences were observed among the strains (Figure 3A). These results indicate that *PAN* plays an important role in early appressorium formation. The delayed appressorium formation in the *PAN* deletion mutants suggests a possible defect in appressorium maturation.

To test this hypothesis, we measured the turgor pressure of appressoria from these strains using exogenous glycerol solutions at concentrations of 1, 2, and 4 M. As expected, the proportion of collapsed appressoria (AP) was significantly higher in Δ*pan2* and Δ*pan3* than in WT, Δ*pan2*/*PAN2*, and Δ*pan3*/*PAN3*, but lower than in Δ*pan2*Δ*pan3* at each glycerol concentration (Figure 3B,C). These findings indicate that both Pan2 and Pan3 are required for appressorium maturation.

Given the role of Pan proteins in appressorium maturation, we hypothesized that glycogen and lipid utilization might be impaired during the transition from conidia to appressoria in the mutants. At 12 h after inoculation on hydrophobic slides, the percentage of WT appressoria stained with KI/I_2_ was significantly lower than that of the *PAN* deletion mutants. By 18 hpi, Δ*pan2* and Δ*pan3* appressoria containing glycogen were noticeably more abundant than those of WT, though fewer than in Δ*pan2*Δ*pan3* (Figure 3D,E). These results suggest that glycogen utilization is disrupted in the *PAN* deletion mutants.

Further lipid staining assays using Nile red revealed that at both 12 and 18 hpi, lipid‐containing appressoria were significantly less common in WT than in Δ*pan2*, Δ*pan3*, or Δ*pan2*Δ*pan3*. Notably, the percentage of stained appressoria in Δ*pan2*Δ*pan3* was higher than in either single mutant (Figure 3F,G), indicating that lipid metabolism is also impaired in the absence of *PAN* function.

Together, these results demonstrate that both Pan2 and Pan3 are essential for appressorium maturation by regulating glycogen and lipid utilization.

### 2.5 Autophagy is Impaired in the Absence of the Pan2‐Pan3 Complex

Impairment of autophagy disrupts the breakdown and utilization of materials such as nuclei, lipids, and glycogen in aged hyphae and conidia. This leads to hindered appressorial development, inadequate turgor pressure generation, and diminished infectivity in host plants [16]. Given the phenotypic similarities with the previously described Δ*pan2*Δ*pan3* mutant, we hypothesize that the Pan complex may also participate in autophagy.

To test this hypothesis, a vector expressing GFP‐Atg8 was constructed and introduced into WT, Δ*pan2*, Δ*pan3*, and Δ*pan2*Δ*pan3* strains. The resulting transformants (WT/GFP‐Atg8, Δ*pan2*/GFP‐Atg8, Δ*pan3*/GFP‐Atg8, and Δ*pan2*Δ*pan3*/GFP‐Atg8) were subjected to nitrogen starvation. After 2 h of treatment, by counting vacuoles within individual hyphal cells, over 40% of GFP‐Atg8‐labeled autophagosomes were observed in the vacuoles of WT, whereas less than 20% were detected in the vacuoles of Δ*pan2*/GFP‐Atg8, Δ*pan3*/GFP‐Atg8, or Δ*pan2*Δ*pan3*/GFP‐Atg8. After 5 h, the percentage of vacuolar GFP‐Atg8‐labeled autophagosomes in WT reached approximately 85%, significantly higher than that in Δ*pan2*/GFP‐Atg8 or Δ*pan3*/GFP‐Atg8 (∼50%) and Δ*pan2*Δ*pan3*/GFP‐Atg8 (∼20%) (Figure S7A,B).

We further assessed autophagy levels in these strains by Western blotting. Autophagic activity was evaluated by calculating the ratio of free GFP to the total of free GFP plus GFP‐Atg8 (GFP/[GFP+GFP‐Atg8]). After 2 and 5 h of nitrogen starvation, this ratio was significantly higher in WT/GFP‐Atg8 than in the mutant strains (Figure S7C,D). These results are consistent with the microscopy data and indicate that autophagic flux is blocked in the *PAN* deletion mutants.

We also monitored the appressorial autophagy process by fluorescence microscopy. In the control strain, GFP‐Atg8‐labeled autophagosomes were readily detected in conidia, germ tubes, and immature appressoria at 0, 2, and 4 hpi, respectively. In contrast, autophagosome signals were markedly reduced at corresponding time points in Δ*pan2*, Δ*pan3*, and especially in Δ*pan2*Δ*pan3* (Figure S7E–H). These findings suggest that the Pan complex is essential for autophagosome formation and thus plays a critical role in autophagy.

### 2.6 P‐Body Biogenesis Depends on Pan2‐Pan3 Activity

**FIGURE 4:**
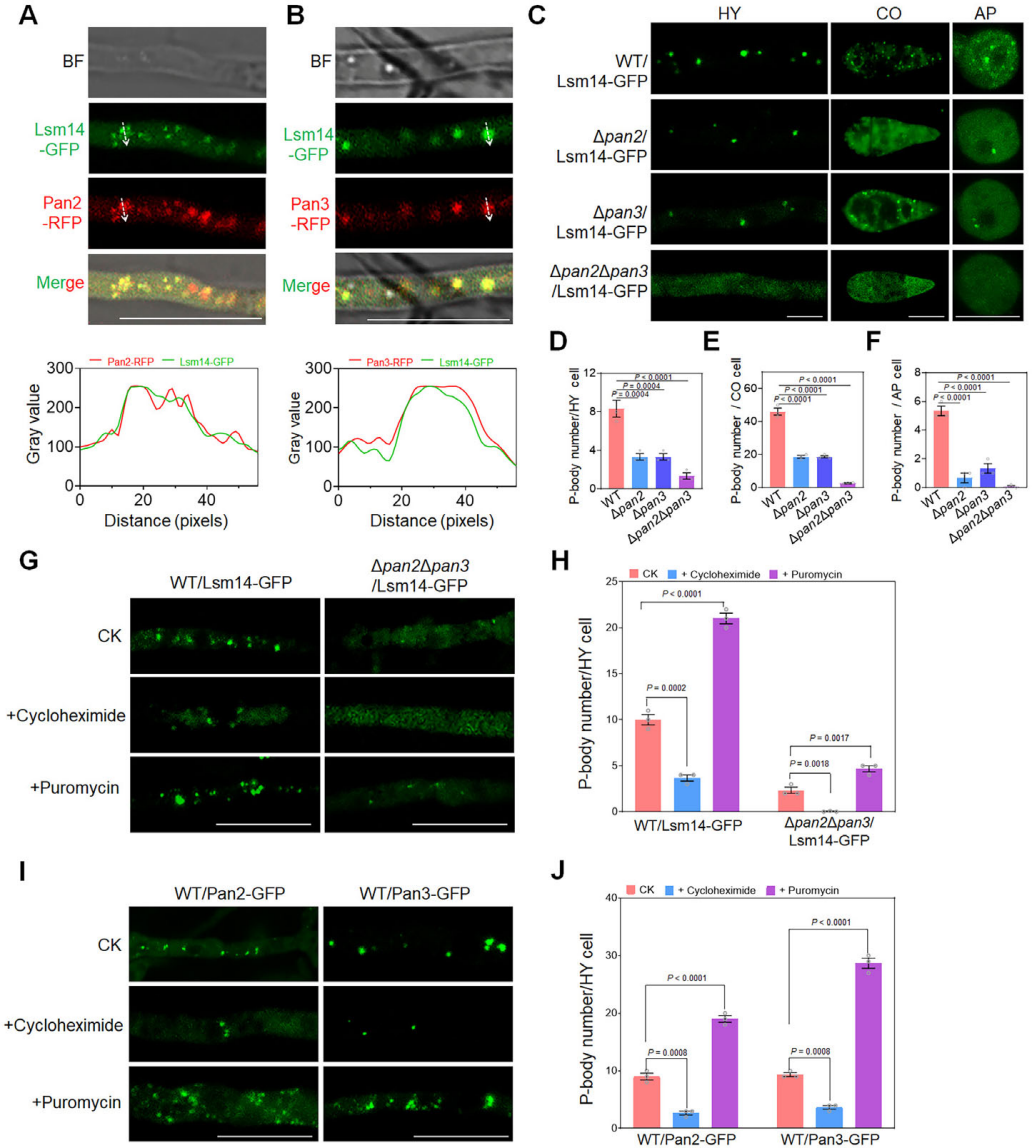
The Pan2‐Pan3 complex regulates P‐body formation. A) Colocalization of Lsm14‐GFP and Pan2‐RFP. B) Colocalization of Lsm14‐GFP and Pan3‐RFP. C) Fluorescence intensity of Lsm14‐GFP in WT, Δ*pan2*, and Δ*pan3*. Scale bar: 20 µm. BF, bright field. D‐F) P‐body numbers per hyphal cell (D), conidial cell (E), and appressorial cell (F) in WT, Δ*pan2*, Δ*pan3*, and Δ*pan2*Δ*pan3* (one‐way ANOVA, Dunnett's test, three biological replicates, error bars = SD). 50 cells of hyphae, conidia, and appressoria were counted per replicate. G) P‐body observation in vegetative hyphae of WT expressing Lsm14‐GFP or Δ*pan2*Δ*pan3* expressing Lsm14‐GFP after treatment of cycloheximide or puromycin. Scale bar: 20 µm. H) P‐body numbers per hyphal cell treated with cycloheximide or puromycin (one‐way ANOVA, Dunnett's test, three biological replicates, error bars = SD). For each replicate, a total of 50 hyphal cells were randomly selected and counted. I) P‐body observation in vegetative hyphae of WT expressing Pan2‐GFP or Pan3‐GFP after treatment of cycloheximide or puromycin. Scale bar: 20 µm. J) P‐body numbers per hyphal cell treated by cycloheximide or puromycin (one‐way ANOVA, Dunnett's test, three biological replicates, error bars = SD). For each replicate, a total of 50 hyphal cells were randomly selected and counted.

P‐bodies (Processing bodies) are membraneless, dynamically assembled organelles in the cytoplasm of eukaryotic cells that serve as sites for mRNA degradation and temporary storage [19]. Given the role of the Pan complex in mRNA adenylation, we hypothesized that both Pan2 and Pan3 localize P‐bodies. To test this, we generated complemented strains expressing Pan2‐RFP in Δ*pan2* and Pan3‐RFP in Δ*pan3*, respectively, and examined their subcellular localization. As expected, both Pan2‐RFP and Pan3‐RFP colocalized with the known P‐body marker Lsm14‐GFP (Figure 4A,B).

We next asked whether the Pan complex influences P‐body formation. In the vegetative hyphae, conidia, and appressoria of WT strain expressing Lsm14‐GFP, we observed intense punctate fluorescence corresponding to P‐bodies. In contrast, Δ*pan2* and Δ*pan3* mutants showed sparse GFP signals, and the double mutant Δ*pan2*Δ*pan3*/Lsm14‐GFP exhibited virtually no punctate fluorescence (Figure 4C–F). These results indicate that the Pan complex localizes to P‐bodies and is required for their formation.

Cycloheximide is a translation inhibitor known to disperse P‐bodies [20, 21], and conversely, puromycin is another translation inhibitor that increases non‐translatable mRNPs and promotes P‐body assembly [21, 22]. WT/Lsm14‐GFP and Δ*pan2*Δ*pan3*/Lsm14‐GFP strains were treated with these two inhibitors, respectively. Cycloheximide treatment significantly reduced the number of punctate P‐bodies in both strains, whereas puromycin treatment markedly increased P‐body formation, confirming that cycloheximide and puromycin indeed suppress and promote P‐body assembly, respectively (Figure 4G,H).

Further, treatment of WT strains expressing Pan2‐RFP or Pan3‐RFP with cycloheximide led to a pronounced decrease in P‐body numbers, visualized by a reduction in Pan2 or Pan3 foci. In contrast, puromycin treatment significantly enhanced P‐body formation in the same strains (Figure 4I,J).

Together, these findings demonstrate that Pan2 and Pan3 are localized to P‐bodies and regulate their biogenesis.

### 2.7 The Pan2‐Pan3 Complex Regulates mRNA Deadenylation of *M. oryzae*

**FIGURE 5:**
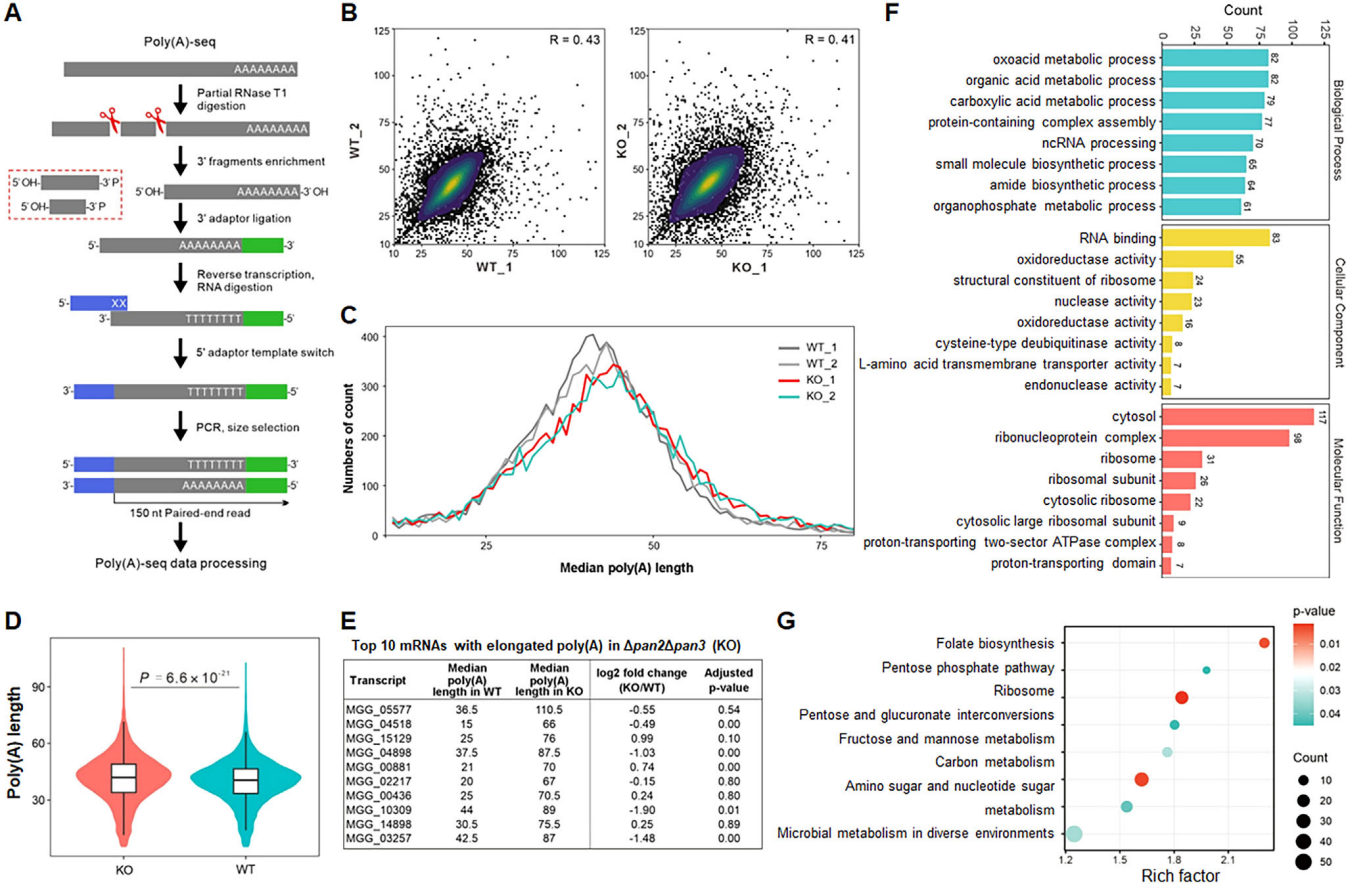
The Pan2‐Pan3 complex regulates mRNA deadenylation in *M. oryzae*. A) Workflow for Poly(A)‐seq library construction. B) Scatter plots of poly(A) lengths in two biological replicates of WT (left) and Δ*pan2*Δ*pan3* (right). C) Median per‐gene poly(A)‐tail length distribution in WT and Δ*pan2*Δ*pan3* appressorial mRNAs. KS test, *p* = 1.0 × 10^−4^. D) Violin plots showing genome‐wide poly(A) tail length distributions in WT and Δ*pan2*Δ*pan3*. Error bars represent SD of two biological replicates. Statistical significance via unpaired *t*‐test. For WT, *n* = 9521; For KO, *n* = 9235. E) Top 10 genes with the most elongated poly(A) tails in Δ*pan2*Δ*pan3* vs. WT. F) GO enrichment analysis of genes with poly(A) tail elongation ≥ 5 nt in Δ*pan2*Δ*pan3*. G) KEGG pathway enrichment of genes with poly(A) tail elongation ≥ 5 nt.

To investigate the role of the Pan2‐Pan3 complex as a deadenylase, we performed poly(A)‐seq—a next‐generation sequencing method for genome‐wide poly(A) tail profiling—to assess the impact on deadenylation across the transcriptome. The workflow of poly(A)‐seq library construction is illustrated in Figure 5A. Data processing and analysis revealed the poly(A) tail length distribution of total RNAs in two biological replicates each of WT (WT_1 and WT_2) and Δ*pan2*Δ*pan3* (KO_1 and KO_2) (Table S1 and Figure S8A). Principal component analysis demonstrated strong reproducibility between the two replicates each for WT and KO samples, along with a clear separation between the WT and Δ*pan2*Δ*pan3* groups (Figure S8B). In *M. oryzae*, poly(A) tails do not exceed 130 nt, with the majority ranging between 20 and 70 nt (Figure 5B).

We further examined the relationship between transcript abundance and median poly(A) tail length. The distribution curve for Δ*pan2*Δ*pan3* was notably shifted to the right compared to that of WT, particularly at the peak region (Figure 5C). This difference was also evident in a violin plot, which confirmed that the average poly(A) tail length was longer in the mutant than in WT (Figure 5D).

Following filtration of the poly(A)‐seq data at *p* ≤ 0.05, a total of 3091 genes were identified, of which 830 had *p*‐values between 0.01 and 0.05. 2531 genes exhibited longer poly(A) tails in Δ*pan2*Δ*pan3* compared to WT, 469 genes had shorter poly(A) tails, and 91 genes showed no difference in poly(A) tail length. The majority of genes demonstrated longer poly(A) tails in Δ*pan2*Δ*pan3* than in WT, which is consistent with our expectations. Considering the risk of false positives and the fact that most length differences clustered within 20–70 nt, we established a threshold of ≥5 nt longer in Δ*pan2*Δ*pan3* compared to WT to define genes with genuinely increased poly(A) tail length. Based on this criterion, 1551 genes were identified as having significantly longer poly(A) tails in Δ*pan2*Δ*pan3*, and these genes were selected for subsequent analysis (Table S2).

Among the top 10 mRNAs with the most elongated poly(A) tails in Δ*pan2*Δ*pan3* vs. WT, MGG_05577 exhibited a median poly(A) tail length of 36.5 nt in WT compared to 110.5 nt in the mutant (Figure 5E). Gene Ontology (GO) analysis of transcripts with significantly elongated poly(A) tails in the mutant revealed enrichment in biological processes such as oxoacid metabolic process, organic acid metabolic process, and ncRNA processing. Within the Cellular Component category, RNA‐binding related terms were most enriched. Additionally, Molecular Function terms were primarily associated with ribosome‐related activities (Figure 5F). Consistent with these findings, KEGG pathway analysis also indicated enrichment for ribosome‐related pathways (Figure 5G). These GO and KEGG results align with the known functions of Pan deadenylases in RNA processing and translation regulation.

Together, our findings demonstrate that the Pan2‐Pan3 complex is essential for mRNA deadenylation in the rice blast fungus.

### 2.8 The Pan2‐Pan3 Complex is Involved in RNA‐Related Processes and Regulates the Expression of Pathogenicity‐Associated Genes

**FIGURE 6:**
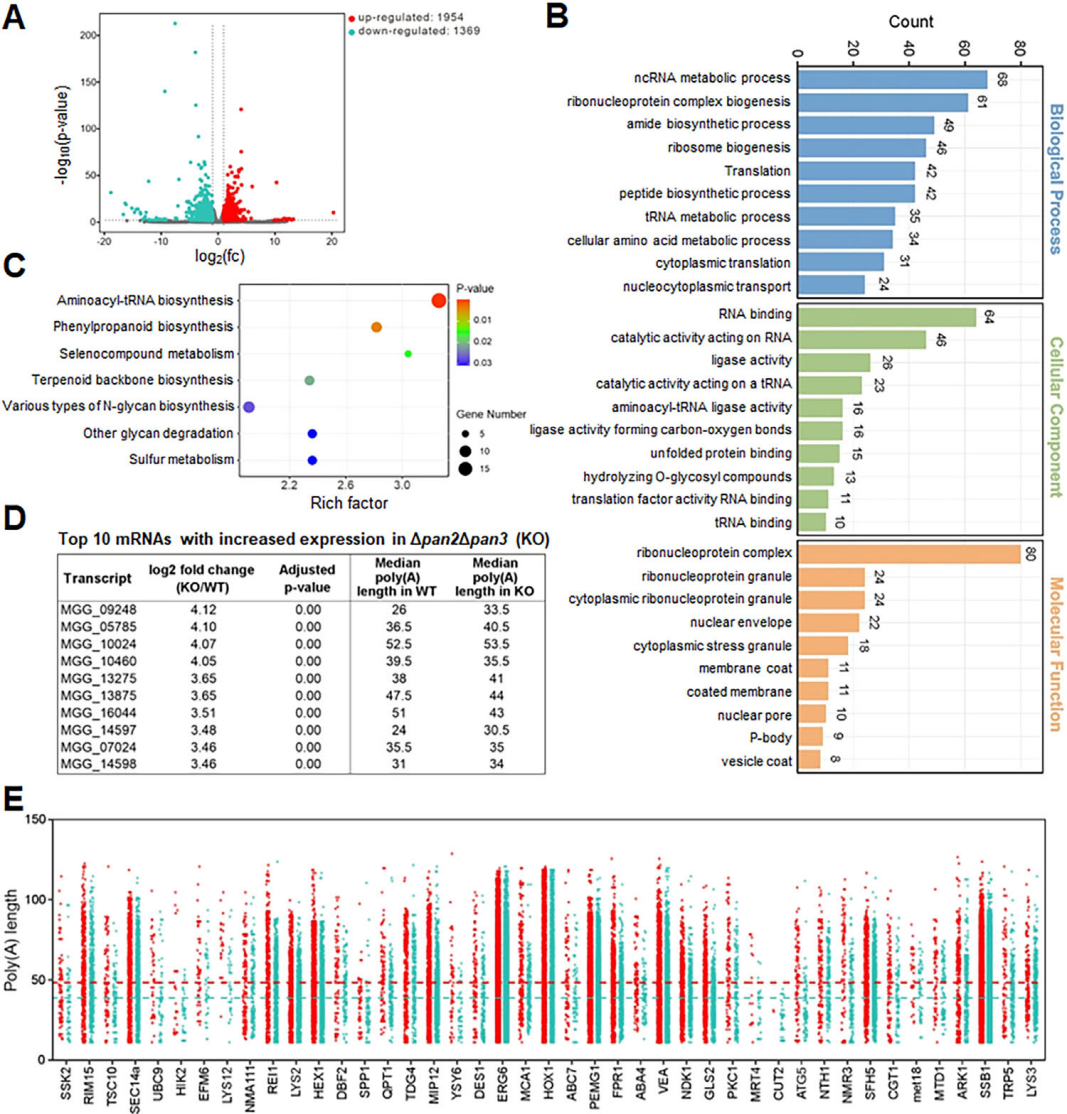
The Pan2‐Pan3 complex participates in RNA‐related pathways and regulates mRNA deadenylation for multiple genes. A) Volcano plot of mRNA expression in Δ*pan2*Δ*pan3* vs. WT. B) GO enrichment of significantly up‐regulated genes in Δ*pan2*Δ*pan3*. C) KEGG pathway analysis of up‐regulated genes in Δ*pan2*Δ*pan3*. D) Top 10 most up‐regulated genes in Δ*pan2*Δ*pan3*. E) Scatter plot of poly(A) tail length distributions for 43 reported genes showing both increased expression and ≥5 nt elongation. The red and green dots represent the poly(A) length of each read for different genes in Δ*pan2*Δ*pan3*, and in WT, respectively. The red and green horizontal dashed line indicates the mean poly(A) length of these 43 genes in Δ*pan2*Δ*pan3* and in WT, respectively.

To further investigate the impact of the Pan complex on RNA expression, we performed RNA sequencing (RNA‐seq) using appressoria from WT and Δ*pan2*Δ*pan3*. A total of 1954 genes were significantly up‐regulated (Fc ≥ 1.5) and 1369 were down‐regulated (Fc ≤ 0.67) in the mutant (Figure 6A and Table S3). Due to the absence of the Pan complex, the deadenylation process is impaired, leading to transient accumulation of cellular mRNAs as they cannot be degraded in a timely manner, manifesting as elevated expression levels in the RNA‐seq data. Therefore, we focused on the 1954 genes showing upregulated expression in the transcriptome. Gene Ontology (GO) analysis of these upregulated genes revealed enrichment in RNA‐related biological processes, including ncRNA metabolic process, ribonucleoprotein complex biogenesis, and tRNA metabolic process. Additionally, molecular functions such as RNA binding, catalytic activity acting on RNA, and aminoacyl‐tRNA ligase activity were enriched. Cellular component terms including ribonucleoprotein complex and P‐body were also identified (Figure 6B). KEGG pathway analysis further indicated enrichment in aminoacyl‐tRNA biosynthesis (Figure 6C). These results suggest that the Pan complex plays an important role in RNA‐related processes, consistent with its function in poly(A) tail deadenylation.

Based on the criteria of FPKM ≥ 1 in RNA‐seq and the presence of a median poly(A) length value detected in poly(A)‐seq, we identified the top ten most significantly upregulated mRNAs in Δ*pan2*Δ*pan3*. Most of these exhibited longer poly(A) tails compared to WT (Figure 6D). Among these, MGG_05785 (designated as *INV1*) has been reported to play significant roles in both pathogenicity and cell wall integrity of *M. oryzae* [23]. Integrated analysis of poly(A)‐seq and RNA‐seq data revealed 390 genes in Δ*pan2*Δ*pan3* that exhibited both elongated poly(A) tails (≥ 5 nt) and significant transcriptional upregulation compared to WT. Among these, 43 were previously characterized genes (Figure 6E), with notably 18 being reported as pathogenicity‐related in *M. oryzae* (Table 1) [24, 25, 26, 27, 28, 29, 30, 31, 32, 33, 34, 35, 36, 37, 38, 39, 40, 41].

**TABLE 1:** A list of reported virulence‐associated genes with significantly elongated poly(A) length and elevated expression in Δ*pan2*Δ*pan3* compared with WT.

Gene_id	Name	Δpoly(A) length^a^	Description	FC^b^	q‐value	Refs.
MGG_09262	*ATG5*	18.5	Autophagy protein 5	3.25	6.4E‐10	[24]
MGG_00970	*UBC9*	18	SUMO‐conjugating enzyme ubc9	1.65	2.1E‐02	[25]
MGG_03148	*TDG4*	15.5	Putative uncharacterized protein	1.73	2.4E‐09	[26]
MGG_04163	*DES1*	14.5	Putative uncharacterized protein	1.65	4.1E‐02	[27]
MGG_09750	*NMR3*	9.5	Diphosphomevalonate decarboxylase	3.20	6.8E‐06	[28]
MGG_08689	*PKC1*	9.5	AGC/PKC protein kinase	2.33	1.8E‐06	[29]
MGG_04626	*MCA1*	9	Metacaspase	2.99	4.0E‐09	[30]
MGG_00345	*RIM15*	9	AGC protein kinase	1.58	2.5E‐02	[31]
MGG_09100	*CUT2*	8.5	Cutinase	6.51	1.7E‐02	[32]
MGG_03028	*QPT1*	8.5	Nicotinate‐nucleotide diphosphorylase	1.68	3.4E‐02	[33]
MGG_08623	*GLS2*	8	Neutral alpha‐glucosidase AB	2.13	7.6E‐04	[34]
MGG_11326	*ARK1*	7.5	NAK protein kinase	1.65	3.6E‐04	[35]
MGG_08622	*NDK1*	7.5	Nucleoside diphosphate kinase	3.22	3.9E‐34	[36]
MGG_08556	*VEA*	7.5	Putative uncharacterized protein	1.63	1.1E‐03	[37]
MGG_02696	*HEX1*	7	Woronin body major protein	1.91	1.8E‐08	[38]
MGG_05307	*PEMG1*	7	Putative uncharacterized protein	2.07	2.9E‐08	[39]
MGG_02611	*LYS2*	6	L‐aminoadipate‐semialdehyde dehydrogenase	3.09	1.3E‐06	[40]
MGG_00183	*SSK2*	5	STE/STE11 protein kinase	1.57	5.5E‐03	[41]

^a^ The difference in poly(A) length obtained by subtracting WT from Δ*pan2*Δ*pan3* in the poly(A)‐seq data.

^b^ The fold change of mRNA abundance in Δ*pan2*Δ*pan3* compared to WT in the RNA‐seq data.

In summary, the Pan complex participates in RNA‐related pathways and regulates mRNA deadenylation for multiple virulence‐associated genes.

### 2.9 The Pan2‐Pan3 Complex Regulates Deadenylation of Three Key mRNAs Involved in Appressorium Function and Virulence of *M. oryzae*

**FIGURE 7:**
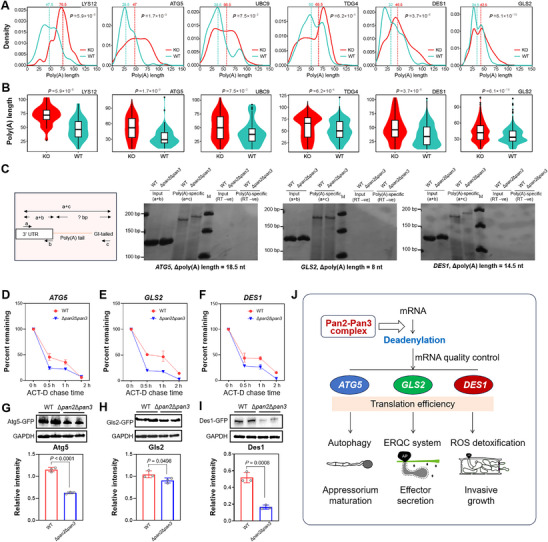
The Pan2‐Pan3 complex regulates deadenylation of key mRNAs essential for virulence. A) Poly(A) length profiles of *LYS12*, *ATG5*, *UBC9*, *TDG4*, *DES1*, and *GLS2* mRNAs in WT and Δ*pan2*Δ*pan3*. B) Violin plots of poly(A) tail lengths for the indicated genes. Error bars represent SD of two biological replicates. Statistical significance via unpaired *t*‐test. C) PCR‐based poly(A) tail length assay for *ATG5*, *GLS2*, and *DES1*. cDNA was synthesized from G/I‐tailed mRNA. Amplification used (a) gene‐specific forward + (b) reverse primers for the 3′ UTR, or (a) forward + (c) universal reverse primer for the tail. Poly(A) length was calculated by subtracting (a + b) product size from (a + c) product size (nt). The validation of each gene was performed with three independent biological replicates. D‐F) mRNA decay rates of *ATG5* (D), *GLS2* (E), and *DES1* (F) after actinomycin D treatment (0.5, 1, and 2 h), measured by RT‐qPCR and normalized to *β‐ACTIN*. Initial level set to 100%. The experiment was performed with three independent biological replicates, error bars = SD. G–I) Western blot analysis of Atg5 (G), Gls2 (H), and Des1 (I) protein levels in WT and Δ*pan2*Δ*pan3*. GAPDH served as loading control. The experiment was performed with three independent biological replicates (one‐way ANOVA, Dunnett's test, error bars = SD). J) Regulatory mechanism of the Pan2‐Pan3 complex in the virulence of *M. oryzae*. The Pan2‐Pan3 complex plays a critical role in mRNA deadenylation and decay, thereby regulating mRNA quality and facilitating the efficient translation of key proteins—such as Atg5, Gls2, and Des1, which are essential for autophagy, ERQC, and ROS detoxification, respectively, and collectively contribute to the promotion of pathogenicity.

Among the reported virulence‐associated genes, we selected five genes (*ATG5*, *UBC9*, *TDG4*, *DES1*, and *GLS2*) that exhibited significantly elongated poly(A) tails and elevated expression in Δ*pan2*Δ*pan3* for further analysis. The poly(A) length distributions in WT and Δ*pan2*Δ*pan3* were visualized using density plots. For each gene, the entire curve for Δ*pan2*Δ*pan3* was shifted to the right compared to that of WT, indicating elongated poly(A) tails in the mutant (Figure 7A). This observation was further supported by violin‐box plots, which confirmed the increased poly(A) length of these genes in Δ*pan2*Δ*pan3* relative to WT (Figure 7B). To further investigate the regulatory role of the Pan complex in virulence, we focused on three genes: *ATG5*, which is involved in autophagy [24]; *GLS2*, which functions in the endoplasmic reticulum quality control (ERQC) system [34]; and *DES1*, which is important for ROS detoxification [27].

To determine whether the poly(A) tail length of these three mRNAs is influenced by the Pan complex, we analyzed poly(A) tail length in appressoria from WT and Δ*pan2*Δ*pan3*. A significant difference in poly(A) tail length was observed between the mutant and WT for all three mRNAs, consistent with the poly(A)‐seq data (Figure 7C). These results indicate that the Pan complex regulates mRNA decay in a poly(A)‐tail‐length–dependent manner.

We next assessed whether the elevated mRNA levels of these three genes correspond to functional translation. Using an actinomycin D chase assay to monitor mRNA decay—by inhibiting RNA polymerase II to block transcription—we measured the stability of *ATG5*, *GLS2*, and *DES1* transcripts. The half‐life of these mRNAs in Δ*pan2*Δ*pan3* was significantly shorter than in WT (Figure 7D–F). The half‐lives of these mRNAs were shorter in Δ*pan2*Δ*pan3* than in WT, indicating their inherent instability. This phenomenon may be attributed to hyper‐adenylation recruiting additional degradation factors that accelerate mRNA decay. This result suggests that the absence of the Pan2‐Pan3 complex promotes the degradation of *ATG5*, *GLS2*, and *DES1* mRNAs.

Furthermore, we examined whether *PAN* deletion affects the protein levels of Atg5, Gls2, and Des1. Constructs expressing N‐terminally GFP‐tagged versions of these proteins (GFP‐Atg5, GFP‐Gls2, GFP‐Des1) were transformed into WT and Δ*pan2*Δ*pan3*. Despite a borderline significant reduction in Gls2 relative abundance (*p* < 0.05)—which may be attributed to a slight elevation in one Δ*pan2*Δ*pan3* replicate—the markedly lower abundance of the fused proteins in Δ*pan2*Δ*pan3* versus WT is consistent with expected regulation by the Pan complex (Figure 7G–I).

Taken together, these results demonstrate that the Pan2‐Pan3 complex controls the deadenylation of *ATG5*, *GLS2*, and *DES1* mRNAs, thereby modulating their expression and function.

## 3 Discussions

The discovery that the Pan2‐Pan3 deadenylase complex orchestrates *M*. *oryzae* virulence reframes mRNA decay as an active architect, rather than a passive consequence, of fungal pathogenesis. By demonstrating that Pan2‐Pan3‐mediated poly(A) tail shortening is indispensable for appressorium maturation, we reveal a previously hidden layer of post‐transcriptional regulation that synchronizes metabolic reprogramming, autophagy, and host penetration. This temporal coordination is not a mere fine‐tuning of gene expression but a gatekeeping mechanism that determines whether the pathogen can transition from surface recognition to invasive growth. The selective stabilization of *ATG5*, *GLS2*, and *DES1* transcripts only after their poly(A) tails are trimmed suggests that deadenylation acts as a molecular timer, ensuring that autophagic flux, ER quality control, and ROS detoxification are activated in precise sequence (Figure 7J). This insight positions Pan2‐Pan3 at the nexus of developmental timing and virulence execution, offering a mechanistic explanation for how hundreds of infection‐related genes are coordinately deployed during the brief window when the fungus breaches host barriers.

A potential limitation of this study is the small sample size (*n* = 2) for poly(A)‐seq. To mitigate potential bias, when identifying genes with differential poly(A) tail lengths, we directly compared all transcript‐level poly(A) counts between the two WT replicates and the two KO replicates with a Student's *t*‐test, rather than comparing gene‐level median values derived from individual transcripts in WT and KO. Additionally, findings were cross‐validated using RNA‐seq and PCR‐based assays to reduce overall risk. Future studies with larger poly(A)‐seq replicates could refine the landscape of Pan2‐Pan3‐regulated transcripts, but our orthogonal validation supports the robustness of key findings.

Beyond the Pan's role in fungal biology, our study illuminates a fundamental principle of eukaryotic RNA homeostasis. The observation that Pan2 and Pan3 localize P‐bodies and modulate their biogenesis extends the functional repertoire of these membraneless organelles from mRNA storage and decay to the active sculpting of developmental fate. The parallels between Pan2‐Pan3 functions in *M. oryzae* and its roles in mammalian stress granules [21] suggest an ancient, conserved logic by which cells use localized deadenylation to partition mRNAs into distinct functional pools. This cross‐kingdom conservation positions the rice blast fungus as a genetically tractable model to dissect how RNA metabolism within phase‐separated compartments dictates rapid cellular differentiation under environmental pressure.

From an applied perspective, the identification of Pan2‐Pan3 as a virulent linchpin offers a transformative target for crop protection. Unlike conventional fungicides that inhibit enzymatic steps common to both pathogen and host, targeting Pan2‐Pan3 exploits an Achilles’ heel unique to the pathogen. The absence of the complex in plants, combined with its essential role in infection, provides a high therapeutic index for small‐molecule inhibitors that block deadenylation without affecting host physiology. Our demonstration that Pan2‐Pan3 disruption collapses turgor‐driven penetration and invasive growth suggests that chemical modulation of mRNA decay could achieve durable disease control while circumventing the resistance mechanisms that plague current antifungal strategies. Thus, this work not only deepens our understanding of how RNA metabolism shapes pathogenicity but also charts a rational course toward next‐generation, sustainable crop protection.

The mechanistic insights gained here also force a reconsideration of how we annotate virulence factors. Traditionally, genes whose deletion attenuates pathogenicity are catalogued as “virulence factors” without distinguishing whether they act upstream as master regulators or downstream as terminal effectors. Our data reveal that Pan2 and Pan3 sit at the apex of a regulatory hierarchy: by controlling poly(A) tail length, they indirectly dictate the abundance of dozens of proteins that execute penetration, effector secretion, and host immune evasion. This raises the possibility that many previously identified virulence genes are, in fact, passengers whose expression is contingent on a handful of RNA‐binding [42, 43] or RNA‐modifying master switches [44, 45]. Systematic profiling of poly(A) tail dynamics across fungal pathogens could therefore uncover a hidden network of post‐transcriptional regulators that serve as “meta‐virulence factors,” offering a more parsimonious framework for understanding pathogenicity.

Finally, the evolutionary implications of Pan2‐Pan3 conservation across fungi but absence in plants invite speculation about the arms race between hosts and pathogens. Plants have evolved sophisticated RNA surveillance systems, including host‐induced gene silencing [46, 47] and NLR‐mediated recognition of pathogen effectors [48, 49], yet they lack the Pan2‐Pan3 machinery. This asymmetry suggests that plants may have deliberately forfeited deadenylation control to prevent pathogens from co‐opting it, or conversely, that fungi have retained Pan2‐Pan3 precisely to outmaneuver host RNA‐based defenses. Comparative genomics of plant‐associated fungi versus non‐pathogenic relatives could test whether Pan2‐Pan3 has undergone adaptive bursts co‐incident with host jumps, thereby illuminating how RNA decay pathways are sculpted by the evolutionary pressures of pathogenesis.

In closing, this study establishes the Pan2‐Pan3 deadenylase complex as a central, previously unrecognized command node that converts environmental cues into precisely timed gene expression programs essential for *M. oryzae* infection. By integrating mechanistic biochemistry, cell biology, and plant pathology, we reveal how a single post‐transcriptional checkpoint can coordinate metabolic, autophagic, and stress‐response pathways to dictate the outcome of a globally significant plant‐pathogen interaction. These findings not only redefine the molecular circuitry of fungal virulence but also open a strategic avenue for crop protection that targets RNA decay rather than protein function, promising durable resistance in an era of rapidly evolving pathogens.

## 4 Experimental Section

### 4.1 Strains and Culture Conditions

The *M. oryzae* wild‐type strain P131 [50] was used in this study. All strains were cultured on oatmeal tomato agar (OTA) plates at 28°C. Colony diameters were measured 5 days post‐inoculation (dpi). Conidia were harvested from 7‐day‐old OTA cultures for conidiation assays. For DNA and RNA extraction and transformation experiments, all strains were grown in liquid complete medium (CM) at 28°C. To assess fungal responses to environmental stresses, strains were inoculated on CM plates supplemented with 0.2 mg/mL Congo red (CR; Sigma–Aldrich), 0.1 mg/mL calcofluor white (CFW; Sigma–Aldrich), 0.005% SDS, 0.7 M NaCl, or 1.0 M sorbitol. Colony diameters were measured after 5 days of incubation.

### 4.2 Quantitative Real‐Time PCR Analysis

To analyze the expression patterns of *PAN2* and *PAN3*, total RNA was extracted from the following samples: conidia from OTA plates, germ tubes and appressoria collected from Teflon films (0.2 cm thick, 0.3 cm wide), and invasive hyphae isolated from inoculated barley leaves at 18, 24, and 48 hpi. cDNA was synthesized from the extracted RNA for use as a template in RT‐qPCR. The RT‐qPCR assays were performed using SYBR Green PCR Master Mix (Takara, Dalian, China) on an ABI 7500 real‐time PCR system (Applied Biosystems, Foster City, CA, USA). The *M. oryzae* GAPDH gene was used as an internal reference.

### 4.3 Gene Deletion and Complementation

Gene deletion was performed using the split‐PCR strategy as previously described [51]. Briefly, the hygromycin phosphotransferase gene (*HPT*) was used as a selectable marker to generate recombinant fragments. Transformants were selected on 250 µg/mL hygromycin B (Roche, Indianapolis, IN, USA). For the double deletion of *PAN2* and *PAN3*, the neomycin resistance gene (*NEO*) was used to generate *PAN3* recombinant fragments, which were then transformed into the Δ*pan2* mutant. Transformants were selected using 400 µg/mL G418 (Amresco, Solon, OH, USA). Single and double deletion mutants were verified by PCR with indicated primers. For complementation, a vector containing a 1.5 kb promoter region, the coding sequence of either *PAN2* or *PAN3*, and a 0.5 kb downstream region was transformed into the respective mutant strain. Complemented transformants were selected on CM plates supplemented with 400 µg/mL G418 and confirmed by PCR and phenotypic analysis.

### 4.4 Staining Assays

For hyphal tip staining, hyphae of the indicated strains were grown on coverslips placed at the edge of colonies on OTA plates. Hyphal tips were stained with calcofluor white (CFW) solution (Sigma–Aldrich, St. Louis, MO, USA) for 10 min, washed twice with PBS, and observed under a fluorescence microscope (Ni90). For glycogen staining, conidia were adjusted to 1 × 10^5^ spores/mL and inoculated on hydrophobic plastic cover glasses. Samples were kept moist and incubated in the dark. At indicated timepoints (0, 3, 6, 9, 12, 18 hpi), samples were stained with KI/I_2_ solution (60 mg/mL KI, 10 mg/mL I_2_) for 10 min. For lipid staining, conidial suspensions (1 × 10^5^ spores/mL) were inoculated similarly and stained with Nile red solution (50 mm Tris/maleate buffer, 20 mg/mL polyvinylpyrrolidone, 2.5 µg/mL Nile red, pH 7.5) at the same timepoints. Images were captured using a microscope.

### 4.5 Virulence Test

Four‐week‐old rice seedlings (*Oryza sativa cv*. LTH) and one‐week‐old barley seedlings (*cv*. E9) were used for virulence assays. For spray inoculation, conidia were suspended in 0.025% Tween 20 at 3 × 10^4^ conidia/mL for barley and 1.5 × 10^5^ conidia/mL for rice. Leaves were incubated at 28°C under high humidity for 4 days before evaluation. For infection process observation, 2 × 10^5^ conidia/mL droplets were applied to the abaxial side of barley leaves. Epidermal layers were excised and observed at 24, 30, and 42 hpi under a fluorescence microscope (Ni90). Each inoculation assay was performed with three independent biological replicates.

### 4.6 Protein Modeling

Monomeric 3D structures of Pan2 and Pan3 (AF‐G4MX57‐F1‐model_v4.pdb) were predicted using AlphaFold3 and retrieved from UniProt.

### 4.7 Protein Expression and Purification

The coding sequence of *PAB1* was amplified and cloned into the pET‐28a vector to express a His‐tagged fusion protein for purification. Similarly, the CDS of *PAN2* and *PAN3* were cloned into the pACYCDuet‐1 vector to produce His‐tagged proteins. Transformed *E. coli* Rosetta (DE3) cells were induced with 1 mm IPTG (Sigma–Aldrich) and cultured at 16°C for 10 h. Proteins were purified using nickel (Ni^2^^+^) affinity chromatography (Genscript).

### 4.8 Deadenylation Assays

Deadenylation assays were carried out as previously described [7]. Briefly, model RNA substrates with poly(A) tails of varying lengths were transcribed in vitro. The DNA template was subsequently removed by DNase I digestion, and the RNA products were purified through phenol/chloroform extraction followed by ethanol precipitation. Prior to deadenylation assays, in vitro transcribed RNAs were treated with calf intestinal phosphatase (CIP). Deadenylation reactions were performed at 30°C for 10 min in a buffer containing 50 mm HEPES‐NaOH (pH 7.5), 50 mm potassium acetate, 1 mm magnesium diacetate, 0.1 mg/mL bovine serum albumin, and 1 mm DTT. Poly(A) RNP complexes were reconstituted by mixing RNA (final concentration 50 nm) with Pab1 (final concentration 150 nm) for the 90A‐model substrate (Yaohai Biotechnology Co., LTD, Beijing, China), followed by incubation for 30 min at 4°C. Reactions were initiated by adding the Pan2‐Pan3 complex to a final concentration of 2.5 nm. At specified time points, 5 µL aliquots were collected and the reaction was immediately stopped by adding 5 µL of stop buffer (50 mm EDTA, 0.1% SDS). Proteins were digested with Proteinase K (NEB), and samples were diluted with 30 µL of loading dye containing 10 mm EDTA, 0.1% bromophenol blue, and 0.1% xylene cyanol FF in formamide, then boiled at 95°C for 4 min. Reaction products were separated on a 6% polyacrylamide gel containing 7 M urea. RNA markers with defined poly(A) tail lengths were included for size reference. Gels were stained with SYBR Green II (Solarbio) according to the manufacturer's instructions, followed by five washes with 1 × TBE buffer. RNA bands were visualized using a UV transilluminator.

### 4.9 Autophagosome Observation and Autophagy Detection

Hyphae of strains expressing GFP‐ATG8 were cultured in liquid CM medium at 28°C and 160 rpm for 48 h, then transferred to MM‐N medium and induced for 2 or 5 h. Autophagosomes were visualized by GFP‐ATG8 fluorescence using a TCS SP8 confocal microscope (Leica Microsystems, Mannheim, Germany). The proportion of vacuoles containing GFP signal was quantified per 100 hyphal vacuoles. For autophagy analysis, mycelia were cultured similarly, transferred to MM‐N medium, and harvested after 2 or 5 h. Total protein was extracted using IP lysis buffer (Beyotime Co., LTD, Beijing, China) and analyzed by SDS‐PAGE and western blotting with anti‐GFP antibody (1:5000, Sigma). To monitor autophagosome formation during appressorium development, conidial suspensions were inoculated on hydrophobic surfaces, and ATG8 localization was observed and quantified at 0, 2, and 4 hpi.

### 4.10 P‐body Observation and Chemical Treatment

P‐bodies were labeled with Lsm14‐GFP. The Lsm14‐GFP fusion vector was transformed into WT, Δ*pan2*, and Δ*pan3*. Transformants were imaged using a TCS SP8 confocal microscope. For cycloheximide and puromycin treatments, vegetative hyphae were treated with 50 µg/mL cycloheximide (Sigma–Aldrich) for 10 min or 100 µg/mL puromycin (Sigma–Aldrich) for 15 min before imaging.

### 4.11 Bioinformatics Analysis of RNA‐Seq

RNA libraries were prepared using the Illumina Novaseq 6000 platform (LC‐Bio Technology Co., Ltd, Hangzhou, China). Raw reads were processed with fastp to remove adapters, low‐quality bases, and undetermined sequences. HISAT2 was used to map reads to the *M. oryzae* 70‐15 (v8) reference genome. Expression levels were estimated with StringTie using FPKM. Differential gene expression analysis was performed with DESeq2. Genes with FDR < 0.05 and |log_2_(FC)| ≥ 0.58 were considered differentially expressed. Enrichment analyses of GO terms and KEGG pathways were conducted on DEGs.

### 4.12 Poly(A)‐Seq Library Generation and Sequencing

Poly(A)‐seq was performed as described [52]. Total RNA (5 µg) was digested with RNase T1 (Thermo), and polyadenylated mRNAs were enriched using VAHTS mRNA Capture Beads (Vazyme). Libraries were prepared with 3′ adapter ligation (Vazyme NR801), reverse transcription, and PCR amplification using the SMARTer Stranded RNA‐Seq Kit (Takara). Size‐selected (200–500 bp) libraries were sequenced on an Illumina Novaseq Xplus system (150 bp PE).

### 4.13 Poly(A)‐Seq Data Processing

Poly(A) regions were identified from adapter‐trimmed reads by detecting 9A (5′ end, error rate ≤0.1) and 6A (3′ end, error rate ≤0.2) sequences. Reads with poly(A) <10 nt were discarded. Remaining sequences were mapped to the *M. oryzae* genome using Tophat2 (≤2 mismatches). Uniquely mapped reads were used for poly(A) length estimation, defined as the longest contiguous A‐stretch without ≥5 non‐A residues.

### 4.14 Bioinformatics Analysis of Poly(A)‐Seq

Median poly(A) length was calculated per gene. Genes with median zero (<10 nt) in either group were excluded. To identify genes with differential poly(A) tail lengths, the poly(A) counts for all transcripts of each gene were separately pooled from the two WT replicates and the two KO replicates. The mean values between the WT and KO groups were then compared using a Student's *t*‐test, with a significance threshold of *p* ≤ 0.05 and Δpoly(A) length ≥ 5 nt. Functional annotation was performed with eggNOG‐mapper; enrichment analysis used clusterProfiler in R. Figures were generated with ggplot2.

### 4.15 Actinomycin D Chase Experiment

Mycelia of the WT and Δ*pan2*Δ*pan3* strains were shake‐cultured in CM medium for 36 h, followed by treatment with 10 µg/mL actinomycin D (ACT‐D; GLPBIO) for 0.5, 1, and 2 h at 28°C with shaking (160 rpm). Total RNA was extracted and subjected to RT‐qPCR for *ATG5*, *GLS2*, and *DES1*. Untreated appressoria served as controls. *ACTIN* was used as the reference gene.

### 4.16 Poly(A) Tail‐Length Measurement Assay

Poly(A) tail length was measured using the USB Poly(A) Tail‐Length Assay Kit (Affymetrix). RNA was tailed with guanosine/inosine residues, reverse transcribed, and amplified with gene‐specific and universal primers. PCR products were separated on 8% PAGE and silver‐stained [53]. Poly(A) length was calculated by subtracting the amplified 3′ UTR length from the total product size. The poly(A) tail length was detected by using the USB poly(A) Tail‐Length Assay Kit (76455, Affymetrix, Cleveland, Ohio, USA). Specifically, a limited number of guanosine (G) and inosine (I) residues were added to the 3’‐ends of poly(A)‐containing RNAs mediated by the poly(A) polymerase. The tailed‐RNAs were then converted to cDNA through reverse transcription (RT) using the newly added G/I tails as the priming sites. Further, we used the primer set *ATG5*‐polyA‐F and *ATG5*‐polyA‐R for amplification to generate 3’ UTR fragment of *ATG5* (Taking *ATG5* for example). Meanwhile, we used the primer set *ATG5*‐polyA‐F and the universal reverse primer to generate the poly(A) tail‐length PCR products.

### 4.17 Western Blotting

Mycelia grown in CM for 48 h were ground in liquid nitrogen. Proteins were extracted with lysis buffer (50 mm Tris‐HCl, pH 7.5, 100 mm NaCl, 5 mm EDTA, 1% Triton X‐100, 2 mm PMSF) plus protease inhibitors (Sangon). Lysates were centrifuged (12 000 × *g*, 15 min, 4°C); supernatants were boiled in loading buffer and separated by 10% SDS‐PAGE. Proteins were transferred to PVDF membranes and probed with anti‐GFP (1:5000, Abcam) or anti‐GAPDH (1:5000, Huaan) antibodies.

### 4.18 Statistical Analysis

Data are presented as mean ± SD of ≥3 biological replicates. Statistical significance was assessed using one‐way ANOVA, followed by Dunnett's test, with analyses performed in GraphPad Prism (version 8.0.2).

## Author Contributions

D.C. and X.‐L.C. conceived this study, designed the investigation, wrote the manuscript, and supervised the project. Z.L. conducted most of the experiments. J.F. analyzed the poly (A)‐seq and RNA‐seq data. S.Z. conducted the phenotypic analysis. Z.Q. assessed the protein structure. J.P. participated in the design of the investigation.

## Funding

This work was supported by the National Natural Science Foundation of China (32402342, 32272476), Hubei Provincial Natural Science Foundation of China (2024AFB227), and Central Public‐interest Scientific Institution Basal Research Fund (1630042024018).

## Conflicts of Interest

The authors declare no conflicts of interest.

## Supporting information




**Supporting File 1**: advs73908‐sup‐0001‐SuppMat.docx.

**Supporting File 2**: advs73908‐sup‐0002‐Tables‐S1‐S5.xlsx.

## Data Availability

The data that support the findings of this study are openly available in NCBI‐PRJNA1315190 at https://www.ncbi.nlm.nih.gov/bioproject/?term=PRJNA1315190, reference number 53.
