# Supplementary material for: Pan2‐Pan3 Complex‐Mediated Deadenylation Enforces mRNA Quality Control for Infection of the Rice Blast Fungus
Source: Adv Sci (Weinh). 2026 Jan 20;13(18):e18269. doi: 10.1002/advs.202518269 (PMC13042520; doi:10.1002/advs.202518269)
Supplement: Supplementary file 1 — Supporting File 1: advs73908‐sup‐0001‐SuppMat.docx. [file ADVS-13-e18269-s002.docx]

**Supplementary Figures:** **Lv *et al.* (2025).** **Pan2-Pan3 complex-mediated deadenylation enforces mRNA quality control for infection of the rice blast fungus**

**Supporting Information including Figure S1-S8 and Table S1-S5. Tables S1-S5 are provided in a separate Excel file named "Supplementary Tables".**

**
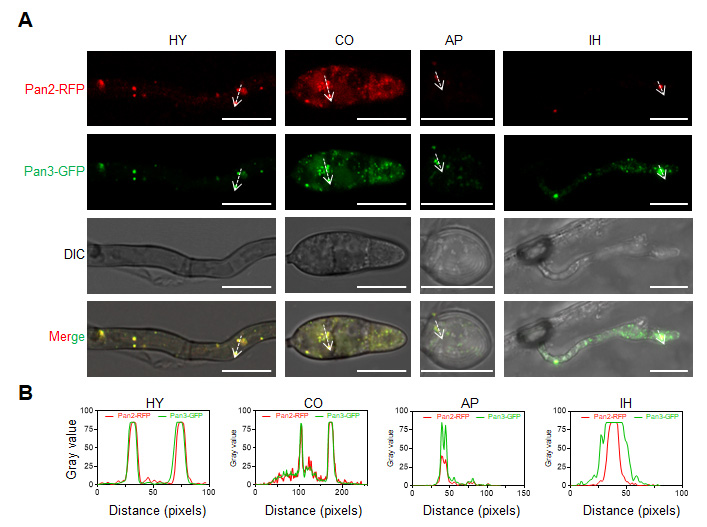
**

**Figure S1.** Colocalization of Pan2 and Pan3 at different developmental stages. A) Pan2-RFP and Pan3-GFP in hyphae (HY), conidia (CO), appressoria (AP), and invasive hyphae (IH). Scale bar: 10 µm. B) Line-scan analyses confirming colocalization of Pan2-RFP and Pan3-GFP.

**
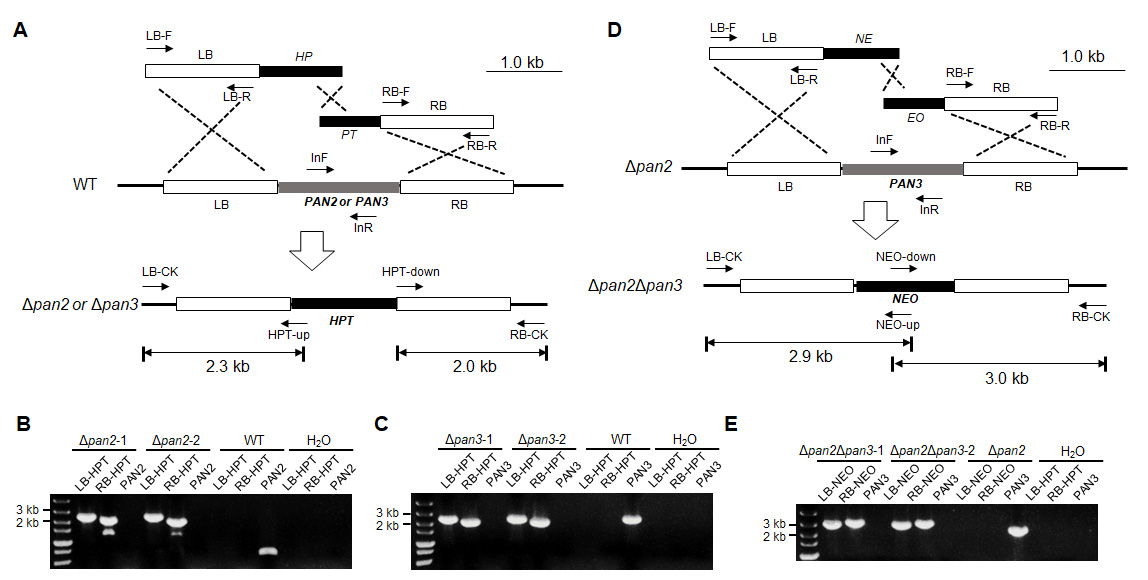
**

**Figure S2.** Generation of Δ*pan2*, Δ*pan3*, and Δ*pan2*Δ*pan3* mutants. A) Strategy for *PAN2* or *PAN3* deletion. B) PCR verification of Δ*pan2* mutants. C) PCR verification of Δ*pan3* mutants. PCR confirmation of two *PAN3* deletion mutants. D) Strategy for double deletion of *PAN2* and *PAN3*. E) PCR verification of Δ*pan2*Δ*pan3* mutants.

**
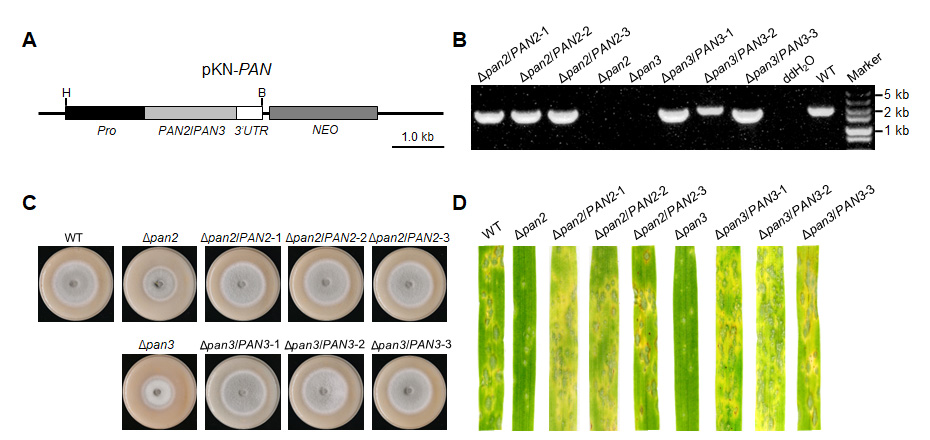
**

**Figure S3.** Complementation of *PAN* deletion mutants. A) Vector design for complementation. B) PCR verification of complementation strains. C) Colony growth of complementation strains. D) Virulence assay on barley leaves.

**
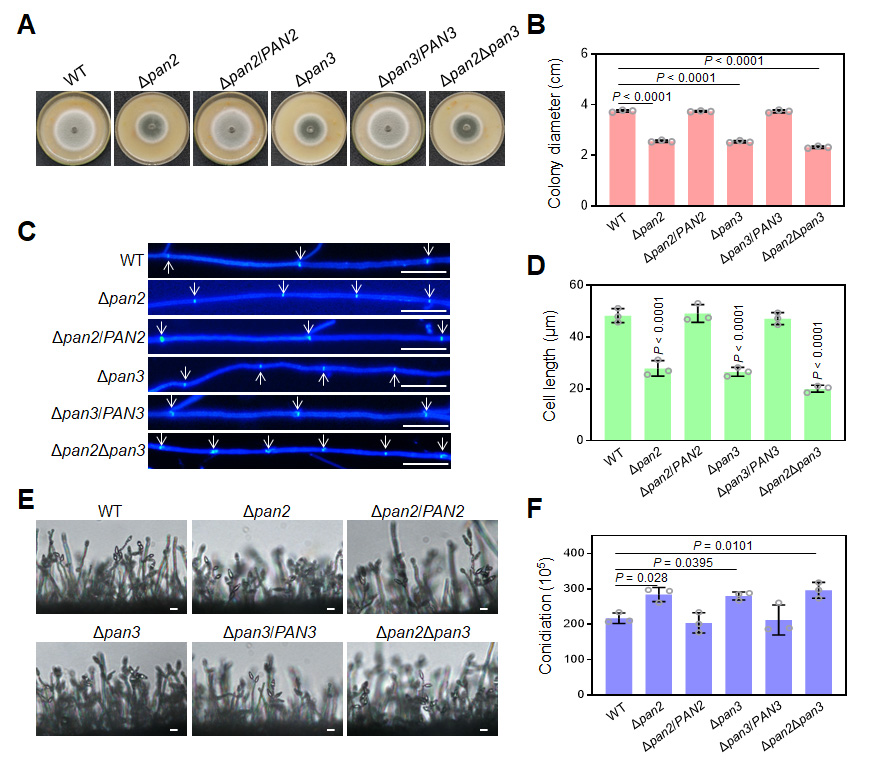
**

**Figure S4.** The Pan2-Pan3 complex is involved in asexual development of *M. oryzae*. A) Colonies grown on OTA for 5 days. B) Colony diameter quantification (one-way ANOVA, Dunnett’s test, n = 3, error bars = SD). C) Hyphal tip staining with CFW. Arrows indicate septa. Scale bar: 20 µm. D) Apical hyphal cell length quantification (one-way ANOVA, Dunnett’s test, three biological replicates, error bars = SD). For each replicate, a total of 50 hyphae were randomly selected and counted. E) Conidiophore morphology. Scale bar: 20 µm. F) Conidiation quantification (one-way ANOVA, Dunnett’s test, three biological replicates, error bars = SD).

**
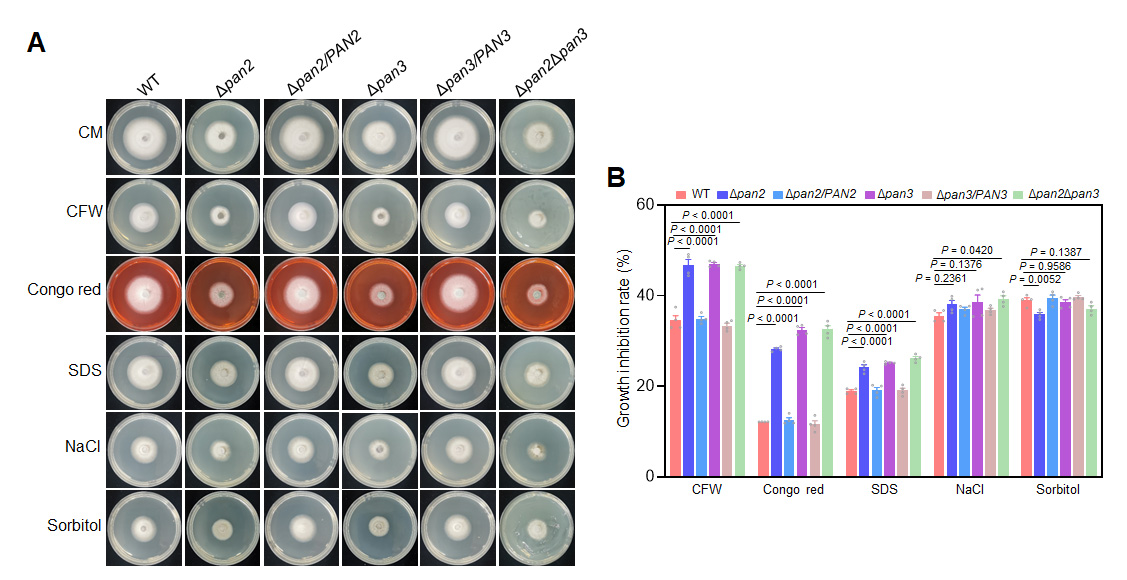
**

**Figure S5.** The Pan2-Pan3 complex is important for cell wall integrity and osmotic stress response. A) Growth on CM supplemented with CFW, Congo red, SDS, sorbitol, or NaCl for 5 days. B) Inhibition rate quantification (one-way ANOVA, Dunnett’s test, n = 4, error bars = SD).

**
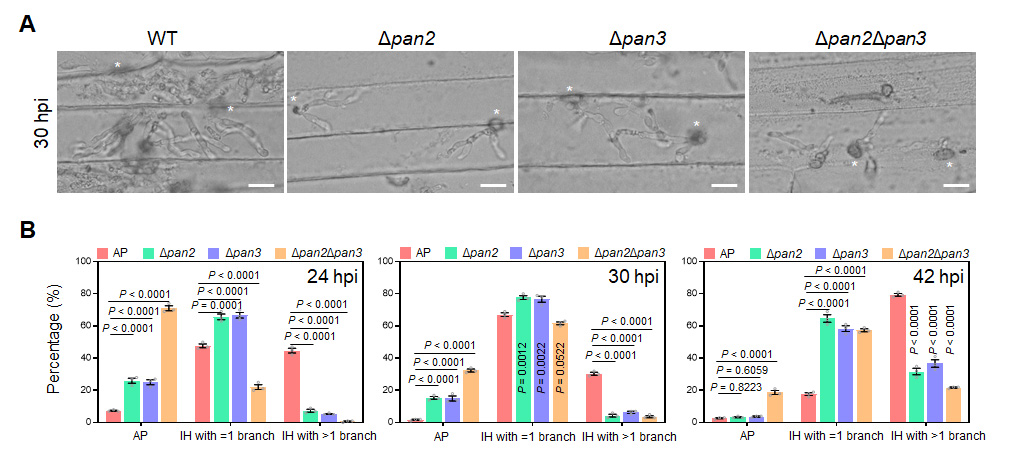
**

**Figure S6.** The Pan2-Pan3 complex is required for invasive growth in barley. A) Invasive growth at 30 hpi. Asterisks indicate appressoria. Scale bar: 20 µm. B) Percentage of AP and branched IH (one-way ANOVA, Dunnett’s test, three biological replicates, error bars = SD). For each replicate, a total of 50 appressoria were randomly selected and counted.

**
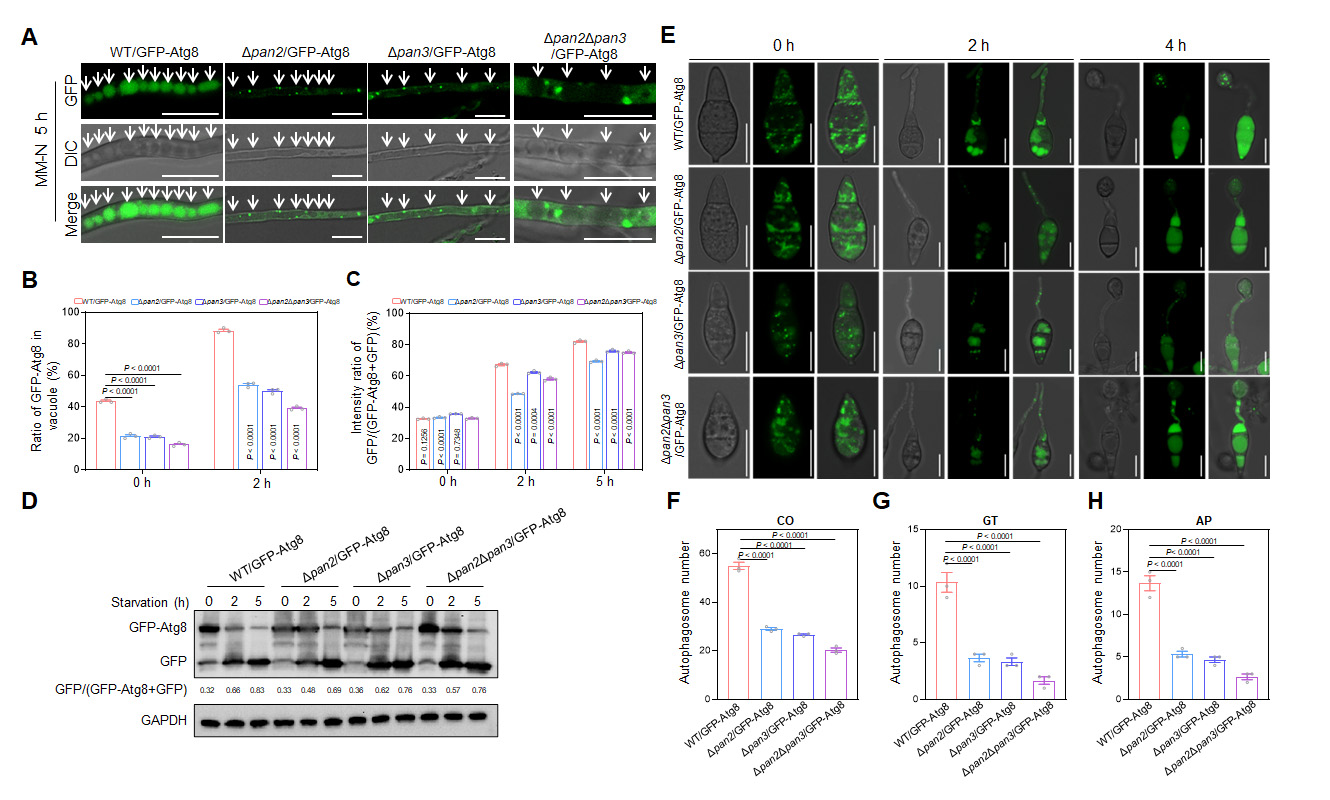
**

**Figure S7.** The Pan2-Pan3 complex affects autophagy and autophagosome formation. A) Localization of GFP-Atg8 after 5 h nitrogen starvation. B) Percentage of vacuoles with GFP-Atg8 signal within individual hyphal cells (one-way ANOVA, Dunnett’s test, three biological replicates, error bars = SD. For each replicate, a total of 50 hyphal vacuoles were counted. C) Immunoblotting of GFP-Atg8 after 0, 2, and 5 h starvation (one-way ANOVA, Dunnett’s test, n = 3, error bars = SD). D) Ratio of free GFP to (GFP-Atg8 + GFP). E) Autophagosome formation during appressorium development at 0, 2, and 4 hpi. Scale bar: 20 µm. F-H) Mean autophagosome number in conidia (CO) (F), germ tubes (GT) (G), and appressoria (AP) (H) (one-way ANOVA, Dunnett’s test, three biological replicates, error bars = SD). 50 cells of conidia, germ tubes, and appressoria were counted per replicate.

**
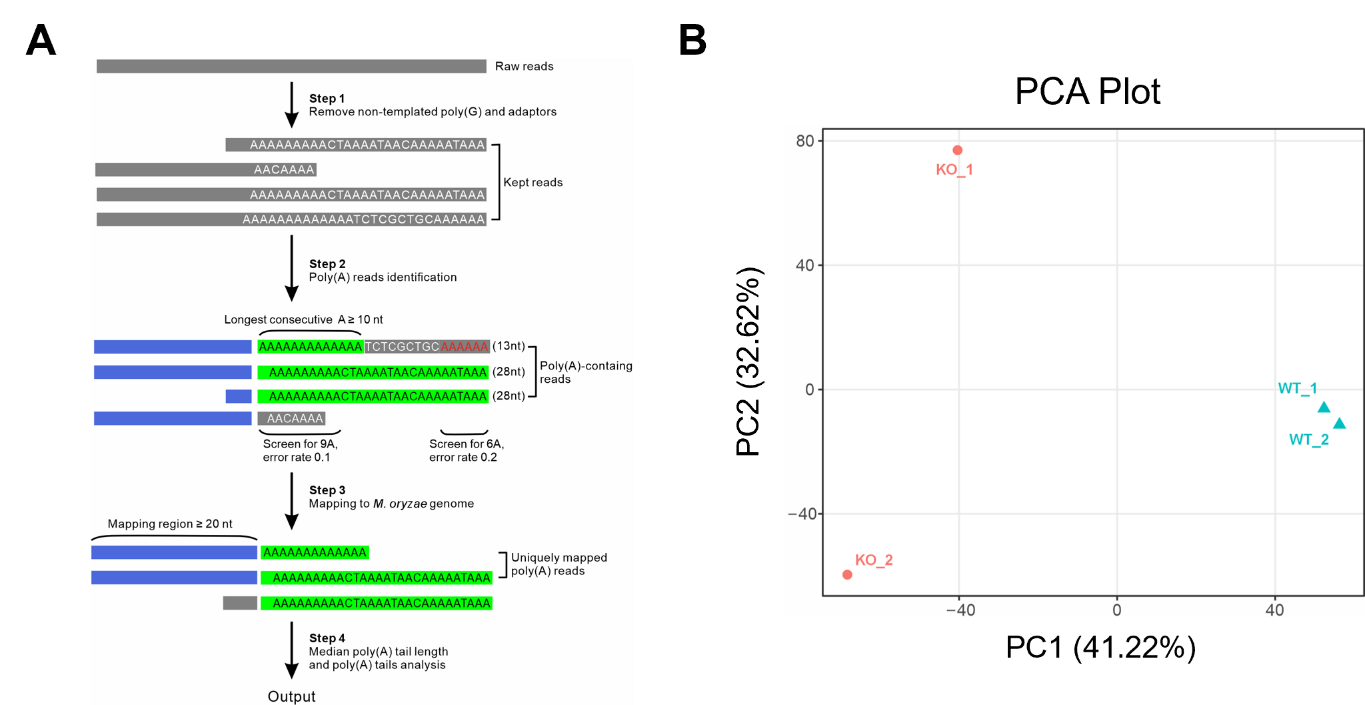
**

**Figure S8.** Flowchart and principal component analysis (PCA) of poly(A)-seq. A) A flowchart illustrating the poly(A)-seq methodology. B) Principal component analysis (PCA) of poly(A)-seq data from wild-type (WT) and the Δ*pan2*Δ*pan3* mutant (KO) samples, respectively.
